# Defining the quality of sediment in the context of the WFD monitoring plans: metal enrichment in two catchments from the north of Portugal

**DOI:** 10.1007/s11368-025-03963-6

**Published:** 2025-02-11

**Authors:** Anabela R. Reis, B. Vieira, Marta Roboredo

**Affiliations:** 1 Department of Geology, School of Life and Environmental Sciences, University of Trás-Os-Montes E Alto Douro (UTAD), Quinta de Prados, 5000-801 Vila Real, Portugal; 2 CGeo - Geosciences Centre, University of Coimbra (Polo II), 3030-790 Coimbra, Portugal; 3 Department of Biology and Environment, School of Life and Environmental Sciences, and CITAB - Centre for Research and Technology of Agro-Environmental and Biological Sciences, CITAB, Inov4Agro, University of Trás-Os-Montes e Alto Douro (UTAD), Quinta de Prados, 5000-801 Vila Real, Portugal; 4 Chemistry Center Vila Real (previous affiliation), University of Trás-Os-Montes e Alto Douro (UTAD), 5000-801 Vila Real, Portugal

**Keywords:** Riverbed sediment, Monitoring plans, Metals, Contamination, Geochemical background, Enrichment factor, Water Framework Directive

## Abstract

**Purpose:**

Riverbed sediment geochemistry provides useful information regarding metal contamination. To integrate sediment quality in river monitoring, within the WFD, the report of sediment quality to water quality managers must be expeditious. This study revisits the metal enrichment concept, applied to sediments from two mountain catchments, as a useful technique in river monitoring.

**Methods:**

Riverbed sediment samples, collected at the end of the Dry and Wet Periods (DP, WP) were analysed for Cd, Cu, Pb, Zn, and Fe in fractions < 2 mm and < 63 µm. The metal enrichment factors (EFs) were referenced to distinct background values: average shale (AS), world rivers suspended sediments (WRSS) and Geochemical Atlas of Portugal (GAP).

**Results:**

Cd, Cu, Pb and Zn contents are higher in the fraction < 63 µm, and at DP. The ranges of variation in fraction < 63 µm are (mg kg^−1^): a) River Vilariça, Cd (5–18 DP; 0.3 WP); Cu (103–341 DP; 22–218 WP); Pb —(24–55 DP; 11–42 WP); Zn (107–241 DP; 54–103 WP); b) River Vizela, —Cd (13–44 DP; 8–41 WP); Cu (267–444 DP; 18–168 WP); Pb —(44–132 DP; 20–42 WP); Zn (141–801 DP; 36–181 WP). Variations in metal contents are influenced by lithological, geomorphological, and microclimatic features, and anthropogenic pressures. EFs are higher when referenced to AS. In the River Vizela, the EFs reveal an enrichment of Cu, Pb and Zn relative to WRSS; Cd registers an enrichment relative to GAP.

**Conclusion:**

Local/regional background, and EFs, are relevant when assessing environmental risks in freshwater systems: low EFs, when associated to natural enrichments, originate values of concern in terms of quality guidelines; high EFs may not imply risk to the fluvial environment. Using the fraction < 63 µm in river monitoring is considered adequate. In dynamic mountain streams, recent sediments and associated contaminants are retained, providing information on possible pollution sources. Identifying metals contamination (or natural enrichment) can help decision-makers to provide solutions for pollution sources.

**Supplementary Information:**

The online version contains supplementary material available at 10.1007/s11368-025-03963-6.

## Introduction

Sediment have a complex and dynamic role in a variety of ecosystem functions and services, in all aspects of the ecosystem’s quality and health services, which can be positive, or negative (Wall 2004; Burkhard et al. 2008; Apitz 2012; Lu et al. 2015; Moore et al. 2017). In order to make progress towards the achievement of Sediment Quality Guidelines (SDGs) targets in the 2030 Agenda, sediment status as an indicator of the ecosystem’s health, as well as an indicator of (sources of) pollution in the upstream drained areas of a catchment, needs to be addressed in river monitoring and management plans.

Through the last decades, there has been an increased awareness of the ecological status of natural ecosystems, followed by the publication of several environmental directives. In Europe, the implementation of the Water Framework Directive in 2000 (WFD, European Commission Directive 2000/60/EC) has led to an increase in research related to the study of ecosystems indicators, and their use as a means to assess their condition along with the processes and mechanisms that drive ecosystem health. Sediment is not specifically addressed in the majority of the directives, in particular in the WFD. However, it is widely accepted that soil and sediment are critical elements in the dynamics of the ecosystems, and knowing and understanding their roles are necessary to meet the environmental quality targets stated for adequate management of ecosystems.

In Portugal, in the ambit of the National Environment and Health Action Plan (PNAAS 2008–2013, Republic of Portugal 2008) the Project Team Soils and Sediments performed a survey on contaminated sites and/or susceptible to anthropogenic and geogenic pollution, based on historical data available for the Portuguese territory. The final report outlined the lack of national guidelines on sediments identical to those provided to other environmental compartments, such as air or water, supported by a specific legislative framework, in order to ensure their global protection against various threats (APA—Portuguese Environmental Agency 2012).

Typically, sediment are considered in environmental pollution studies. However, in the context of river basin monitoring plans (RBMP), sediment management has been considered in only a few major rivers in Europe. Since 2000, a long journey of studies and debate about the relevance of including sediment management in the RBMPs has been promoted by the European multi-stakeholder Sediment Network, SedNet (www.sednet.org) (Förstner 2002; Brils 2020; Owens and Xu 2020). During this period of time, the sediment management was considered for the first time in a RBPM, after a sediment management concept for the Elbe river basin was established (Heininger et al. 2015). In 2022, the WFD Common Implementation Strategy (CIS) Ecological Status (ECOSTAT) working group, with the collaboration of experts from SedNet, drafted the WFD CIS guiding document “Integrated sediment management Guidelines and good practices in the context of the Water Framework Directive” (European Commission 2022). The publication of this document allows Member States to include recommendations on sediment quantity and quality management on the 3rd cycle of the preparation of RBMPs (European Commission 2023). In this context, Brils and Maring (2023) proposed a practical conceptual model for the management of soil-sediment–water ecosystems, which includes a discussion on how the model could support the implementation of the European soil-sediment water policy development in practice.

The WFD CIS document describes how sediment associated contamination can be addressed in a WFD context by applying sediment Environmental Quality Standards (EQSs) (Directive 2008/105/EC and Directive 2013/39/EU), which has already been done by a few Member States to assess the chemical status of water bodies. However, natural (geochemical) background concentrations of contaminants may hinder the achievement of established EQSs. In the same way, in surveillance investigations, as cited by Förstner and Heise (2006), Heise and Förstner (2007) and Förstner et al. (2008), to consider local natural geochemical interferences is indispensable to evaluate the extent of sediment contamination by a specific element. Concerning metals, to distinguish between the human and natural contributions to contents in sediments is a challenge, because of the influence of natural local variability of metals on sediment composition, as well as the fluvial processes involved in its dynamics. Besides, in order to overcome a possible overestimation of metal concentration in unpolluted sediment, it is fundamental to select a proper reference metal concentration (Reimann and de Caritat 2005; Gałuszka 2007; Dung et al. 2013; Sakan et al. 2015; Matys Grygar and Popelka 2016; Lipp et al. 2020; Nawrot et al. 2021).

For the effective integration of sediment quality in the monitoring plans, the analysis of sediment quality, i.e. contaminated or not contaminated, has to be relatively expeditious, and results should be presented in an easy reading and inter-comparable form. Regarding the evaluation of sediment pollution by metal(oid)s, this study revisited the use of Enrichment Factor (EF), which has been commonly used to quantify the enrichment and to distinguish the anthropogenic influence of metal contents in sediment. The use of EFs is, however, controversial, mainly related to a mechanistic application, without addressing fundamental issues, like grain-size control of sediment composition, and appropriate application of a geochemical background of metal contents (Reimann and de Caritat 2000, 2005; Matys Grygar and Popelka 2016; Tůmová et al. 2019).

The data required for the calculation of enrichment of metals in sediments are: (1) the chemical analysis of sediment samples (total or pseudo-total contents, or geochemical fractionation with the determination of contents in the labile phases); and (2) the reference, or background, value for each metal(oid) under study, which refers to its natural content without influence of anthropogenic pollution (Reimann and Garrett 2005). It is common the use of average trace element concentration in: (a) upper continental crust (UCC) (the most common) (Rudnick and Gao 2014); (b) river suspended sediment (Viers et al. 2009; Meybeck 2013; Bravard 2018; Dendievel et al. 2022); (c) average shale (Martin and Meybeck 1979); (d) local pristine sediments, only considered in a few studies (N’guessan et al. 2009; Tapia et al. 2012; Matys Grygar and Popelka 2016). Collecting pristine sediments in mountainous catchments, even in moderately polluted areas, can be a challenge, requiring knowledge of geology and fluvial geomorphology. The reference value (background) commonly used to calculate the enrichment of metals in sediment, is the mean elemental contents of upper continental crust (UCC). The ratio between trace element concentration in suspended sediment from world rivers and in UCC indicates that sediment concentrations present a significant enrichment for all elements (Viers et al. 2009), which is attributed to anthropogenic activities (Nriagu 1979; Nriagu and Pacyna 1988) as well as to specific lithologies. Consequently, the use of UCC as reference material, to consider element contents background, cannot be considered adequate in local and regional river catchments. This approach is valid for larger river basins, where several types of rocks outcrop, but in small-sized catchments hardly reflect the local average bedrock chemical composition (Reimann and De Caritat 2005; N’guessan et al. 2009). Therefore, the classification of the level of contamination in a specific sampling point will be significantly dependent on the background value selected for each metal under consideration.

The topic of this discussion may seem outdated. However, there are still uncertainties and a multitude of procedures for monitoring river contamination levels are applied in practice (e.g. sampling media, analytical procedures) (Mokwe-Ozonzeadi et al. 2019), with the use of mechanistic instead of targeted approaches for each river system, considering its intrinsic physical and chemical characteristics (e.g. lithology, river basin geomorphology and irregularity of river/stream longitudinal profile) (Matys Grygar and Popelka 2016). Recent research addressing sediment contamination often neglects the natural major factors controlling fluvial sediment composition and their influence on the natural chemical background of physical media.

In this context, this study discusses the potential application of the enrichment factor as a tool to report the contamination degree of sediments from two mountainous catchments, within the application of the WFD. The discussion is focused on the geochemical approach to the evaluation of sediment contamination, and the results were interpreted in terms of different background/reference values used in the classification of the quality of the sediment. The selected study areas, located in northern Portugal, are subjected to different environmental pressures, and characterised by different geological settings and microclimatic regimes. On the one hand, this study informs on the state-of-the-art of sediment monitoring, in the context of WFD, in Portugal, which is a member State of the European Union, contributing with knowledge to help decisions of environmental risk managers and decision makers in remediation actions as well as the implementation of a sedimentological network. On the other hand, the discussion about the quantification of metal contents in sediments, from a geochemical perspective, is of broader scientific interest.

## Materials and methods

### Study area

**Fig. 1:**
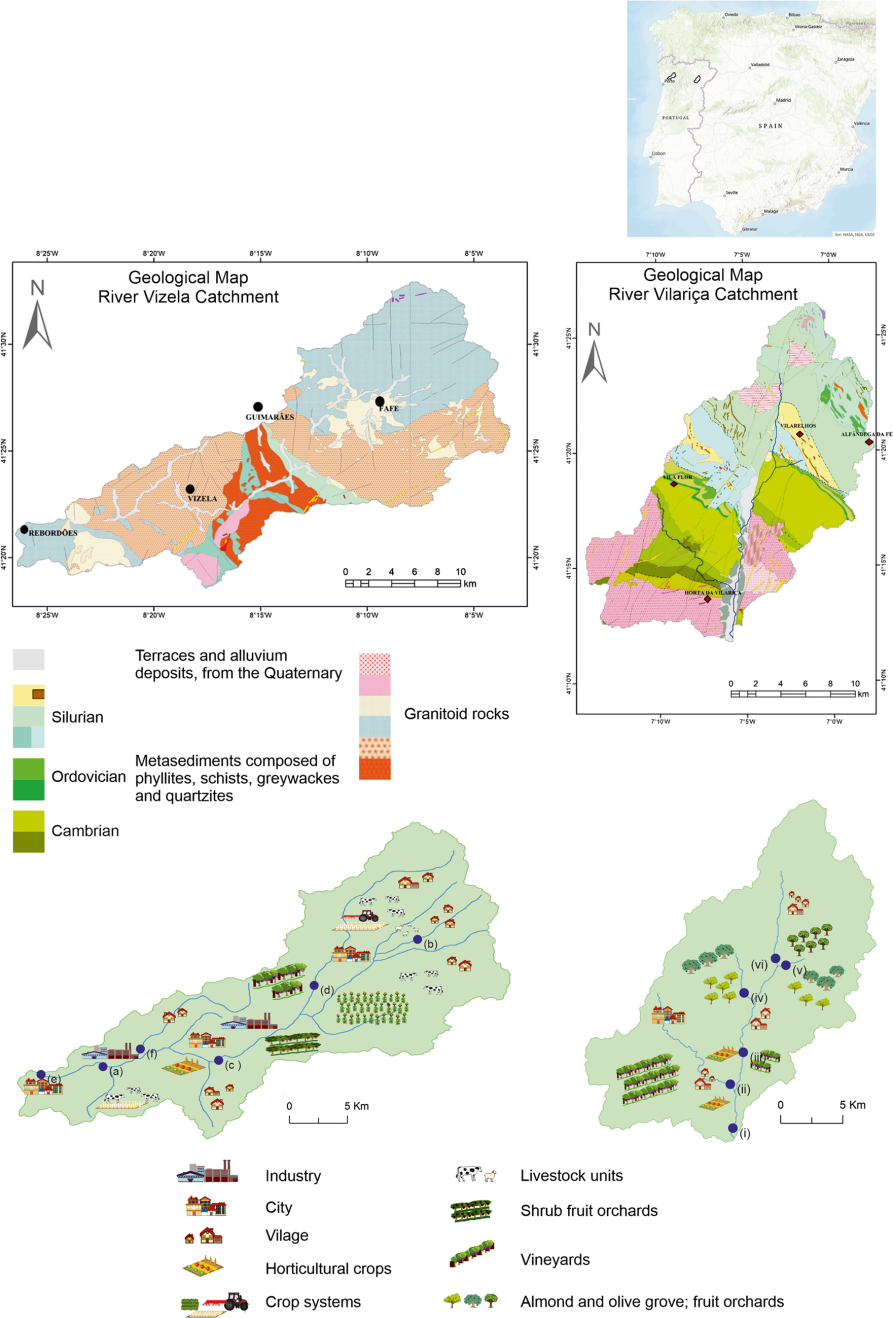
Location of the studied areas in the north of Portugal: **a** River Vizela catchment (NW); **b** River Vilariça catchment (NE); (i) identification of main anthropic pressures and location of sediment sampling stations; (ii) simplified geological map (modified from Geological Map of Portugal, scale 1:200,000, Laboratório Nacional de Energia e Geologia (LNEG), https://geoportal.lneg.pt)

Data used in the present study were obtained from samples collected in two catchments, located in the north of Portugal. The correspondent drainage areas show contrasting features, regarding lithology, geomorphology, microclimate, land use and anthropic pressures (Fig. 1). The River Vizela, located in the northwest, has a catchment area of 342 km^2^, with a population density of 571.6 inhabitants/km^2^ (INE IP 2022), and extending from an altitude of 900 m to 92 m. The bedrock of the drainage area is composed of granitoid rocks, intercepted by a straight band of Silurian metasediments; terraces and alluvium deposits, from the Quaternary, overlay the older lithologies along the main watercourses (Andrade et al. 1986). The microclimate is characterized by temperate and dry summers, with precipitation varying in the range of 1500–2000 mm/year, and mean monthly temperature varying in the range of 7–22 ºC (APA 2021a; IPMA 2011). The land use is mostly forest and shrub vegetation (41%), agriculture (34%), urban and industrial areas (14%), pastures (8%) and semi-natural areas (3%) (Terêncio et al. 2017). Animal manure application to soils predominates in the crop systems, and the livestock units have a strong component of milk production. Local wine production and orchards also occur dispersed in the area. The industrial activities are dominated by the textile sector.

The River Vilariça, located in the northeast, has a catchment area of 324 km^2^, with a population density of 22.8 inhabitants/km^2^ (INE IP 2022), and extending from an altitude of 1186 m to 110 m. In the downstream part of the basin the river drains a large valley of bottom flat, of tectonic origin (Pereira and Pereira 2020). The geology comprises: in the northern part, Paleozoic metasediments composed of phyllites, schists, greywackes and quartzites (Silva et al. 1989); in the southern part of the basin granites outcrop. Locally, Quaternary deposits cover the crystalline rocks (Cunha and Pereira 2000), with major expression southward. The microclimate is characterized by dry and hot summers, with annual precipitation varying in the range of 400 to 800 mm/year, and the precipitation events distributed mostly between the autumn and early spring (APA 2012a). The mean monthly temperature varies between 5 ºC and 25 ºC (APA 2012b; IPMA 2011). The land use is mainly agriculture (42%) and forest (25%), and semi-natural areas (31%), with scattered urban settlements (Santos et al. 2021); the industrial activity is low. The agricultural production is focused on olive groves, vineyards, fruit orchards and horticultural crops.

### Sediment sampling and sample preparation

Six sampling sites were selected in each of the studied catchments, in depositional sections of the river channel where bottom deposits, at shallow water depths, are in active exchange with suspended material. Samples were collected at the end of the Dry Period (DP), in September–October 2016, and at the end of the Wet Period (WP), in April 2017, to obtain information about the variation of the granulometry of deposited sediments in low and high flow conditions, and related pH and organic metal contents. At each sampling site, freshly deposited sediment samples, riverbed oxic layer (0–2 cm), were collected manually, with a stainless-steel spoon (Mudroch and Azcue 1995; Parker et al. 2007). Random sediment samples were collected in a cross-sectional line to the river channel and mixed to obtain a representative sample, of at least 3 kg. The samples, stored and adequately sealed in plastic bags, were transported to the laboratory in thermos boxes and stored at 4 °C, prior to analysis. In the laboratory, the separation of the fractions < 2 mm and < 63 µm was performed by wet sieving with ultra-pure water. After centrifugation, the settled sediment was oven-dried at 35 °C, which is considered to not affect the results of contaminant contents (Kralik 1999; Förstner 2004). Aliquots for the chemical analysis were then taken according to quartering method. All the apparatus used in the sample handling were soaked in 10% HCl solution, followed by a 10% HNO_3_ solution, and rinsed with ultra-pure water.

### Analytical procedure

The particle size analysis of the sediment granulometric fraction < 2 mm, with the determination of the percentages of sand, silt and clay, was carried out on a Skalar Robotic Analyzer SP2000 with the addition of pyrophosphate solution. The previous removal of organic matter, with hydrogen peroxide, and carbonates, with HCl, was carried out in a Skalar Robotic Analyzer. The pH of sediment samples was measured in water (pH-H_2_O) by using a 1:2.5 sediment/solution ratio (Schofield and Taylor 1955). Total C was determined after dry combustion with near infrared detection using an elemental TOC analyzer (Primac SC, Skalar Analytical B.V., Breda, Netherlands). The effective cation exchange capacity ECEC (sum of pH 7.0 ammonium acetate extractable Ca, Mg, K, and Na plus exchangeable acidity extracted by 1 molL^−1^ KCl) was determined following the methodology described by Houba et al. (1995).

The Cd, Cu, Pb, and Zn contents, in the sediment size fractions < 2 mm and < 63 µm, were assessed through the digestion of sediment with hydrogen peroxide and aqua regia (HNO_3_ + HCl), in a high‐pressure microwave digestion unit (Anton Paar Multiwave PRO), following the US EPA Method 3051A (USEPA 2007). The extracts were analysed by optical emission spectroscopy with inductive plasma source (ICP‐OES, Perkin‐Elmer OPTIMA 8300). Blank extractions (i.e. without sediment), were carried through the complete procedure using the same reagents. Laboratory replicates were used to assess the analytical process. To ensure accuracy, an in-house reference material was used. The precision of the measurements was about ± 5%.

The *aqua regia* digestion procedure allows to have information on metal contents in chemical forms of environmental concern, which are associated with non-silicate minerals (Förstner and Salomons 1980). It provides better chances to detect “human impact” and carry out risk analysis (Reimann 2022). The European Commission, in its Guidance Document nº 25 (European Commission 2010), recommends the use of less aggressive acid mixtures (namely *aqua regia*) for surface waters, by ensuring that the final detection technique provides low detection limits. Besides, this digestion procedure was followed by the IGCP Project 259 ‘‘International geochemical mapping’’ (Darnley and Garrett 1990; Darnley et al. 1995), in which the Low-density geochemical mapping in Portugal was integrated (Ferreira et al. 2001).

### Enrichment Factor (EF)

The single element enrichment factor (EF) was selected to assess metal enrichment in sediments, and express the results in a categorized format. Classifications, as long as they are well designed, in addition to allowing data to be presented in a more objective way, allow for easier comparison with similar data from other areas. The EF was determined using the equation (Buat-Menerd and Chesselt 1979, *in* Zhao et al. 2012):

1 $$EF=\frac{{\left(\frac{X}{N}\right)}_{sample}}{{\left(\frac{X}{N}\right)}_{reference}}$$

where ${\left(X/N\right)}_{sample}$ corresponds to the ratio between the content of the metal and the content of the normalizing element in the sample under study; and ${\left(X/N\right)}_{reference}$ corresponds to the ratio between the content of the metal and the normalizer element content in the reference material.

In this study, it was decided to use the conservative element Fe as a normalising element, following the theoretical considerations presented by Matys Grygar and Popelka (2016). Normalising the metal contents allows the correction of any remaining geochemical differences in sediment composition after sieving, given the lithological context and the granulometry of the sediments. The reference materials considered were the composition of: a) average shale (Turekian and Wedepohl, *in* Salomons and Forstner 1984); b) world rivers suspended sediments (Viers et al. 2009); c) stream sediments from the low-density geochemical survey of Portugal (Ferreira 2000; Ferreira et al. 2001) (Table 1). The latter reference values were taken from the geochemical maps produced on data from a sampling density of 1/135 km^2^ (maps were prepared by interpolation into a 22 km regular grid performed by kriging). For each sampling point of this study the correspondent reference value in the elemental maps was considered. On one hand, the average shale composition can be considered as a proxy for sediments of finer grain size, enriched in clay minerals (Reimann and de Caritat 2000). Moreover, in the geochemical cycles, elements undergo natural chemical fractionation, together with an increase in finer particles, resulting in a natural enrichment of heavy metals relative to conservative elements. On the other hand, regional geochemical mapping provides the available multi-element database documenting the surface environment at the time of sampling, in the late 1990s.

**Table 1:** Metal contents (mg kg^−^1) in the reference materials considered to calculate the Enrichment Factors: average shale; suspended sediments of World Rivers; active stream sediments from the low-density geochemical survey of Portugal (ranges of variation)

		Cd	Cu	Pb	Zn	Fe
Average shale (AS) (Turekian and Wedepohl 1961, in Salomons and Forstner 1984)		0.22	45	20	95	47,000
Suspended sediment of World Rivers (WRSS) (Viers et al. 2009)		1.55	76	61	208	58,100
Geochemical Atlas of Portugal (riverbed sediments) (GAP (Ferreira 2000)	River Vilariça	0.20-0.40	25-56	20-47	62-213	24,900-40,500
	River Vizela	0.20	35-48	27-47	108-203	22,600-36,800
Upper continental crust (UCC) Rudnick and Gao (2014)		0.60	35.7	35	123	46,700
Sediments Quality Guidelines, USEPA (SQGs) PEL (Probable Effect Level) (MacDonald et al. 2000)		3.53	197	91.3	315	-

Values of Upper continental crust and Sediment Quality Guidelines – Probable Effect Level (SQG-PEL) are also shown for comparison

The average composition of the Earth's crust was not used as reference material for the reasons already addressed in the introduction section. The use of local background was not possible to consider, because of the reduced number of sampling sites in each basin and the lack of samples that could be considered pristine sediments. Nevertheless, as stated/discussed/argued by Reimann and de Caritat (2005), as well as by Matys Grygar and Popelka (2016), the regional chemical composition of Earth’s surface is not only controlled by lithology; other factors like climate (temperature, precipitation), landscape (elevation, slope, orientation), vegetation, bioproductivity and distance to coast influence the distribution of chemical elements.

There is no recognised categorisation of degree of enrichment based on the EF methodology. In general, it is considered that EF < 2 reflects the natural variability of the sample mineralogical composition, and above 2 an enrichment is supposed to exist (Sutherland 2000; Zhao et al 2012). Several authors consider that when using local geochemical background as reference material the threshold of EF is 1.5 (Roussiez et al. 2005; N’guessan et al. 2009), or 1.0 (Szefer et al. 1999; Devesa-Rey et al 2011; Nawrot et al. 2021). The following categorization was considered, after Sutherland (2000) and Buat-Menerd and Chesselt (1979, in Zhao et al. 2012): 1) EF < 2 - depletion to minimal enrichment; 2) 2 > EF < 5 - moderate enrichment; 3) 5 > EF < 20 - significant enrichment; 4) 20 > EF < 40 - Very highly enriched; and 5) EF > 40 - extremely enriched.

### Data analysis

To investigate the correlations between the contents of metals and the properties of sediments, a Principal Component Analysis (PCA) was carried out on the dataset relative to sediment granulometric fraction < 2 mm. This multivariate statistical technique has been applied in environmental studies related to quality of sediment compartment in fluvial systems (e.g. van der Perk and Vilches 2020; Miranda et al. 2021a, 2022b). The STATISTICA software was used in the statistical analysis. The PCA was performed on the correlation matrix of non-normalised data (Jolliffe 2002; Bispo and Marques 2023).

## Results

### Sediment particle size analysis, pH, organic matter and effective cation exchange capacity

**Fig. 2:**
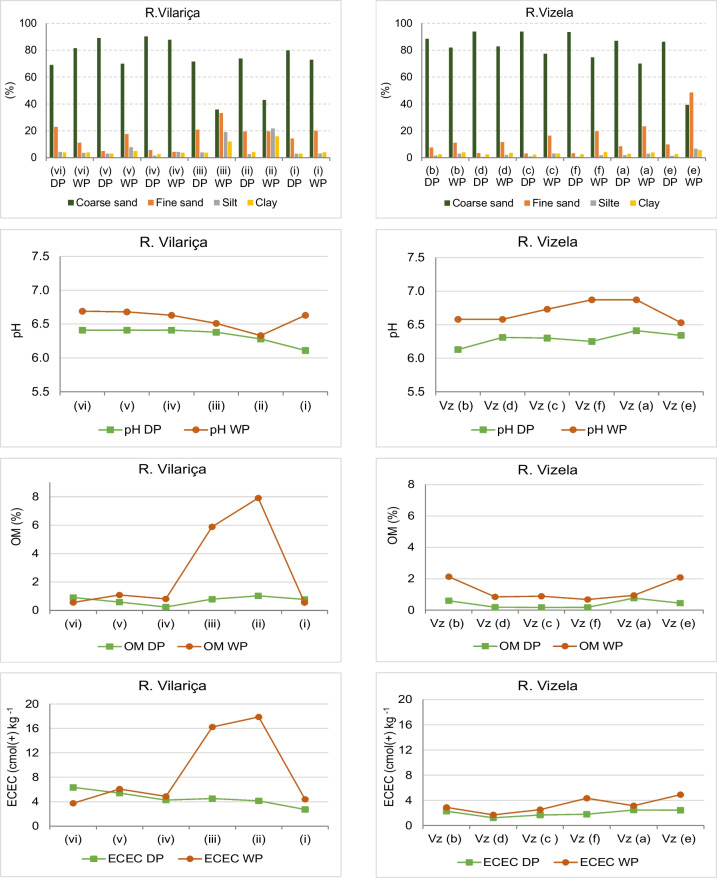
Spatial and temporal variation of sediment grain size (coarse sand, fine sand, silt, clay, %), pH, organic matter (OM, %), and effective cation exchange capacity (ECEC, cmol( +) kg-1) in the granulometric fraction < 2 mm (sample site is indicated from upstream to downstream; DP – Dry Period; WP – Wet Period)

Sediment are composed essentially of sand size particles (> 70%) (Table 2). Silt and clay granulometric classes represented 3–12% of the < 2 mm sediment fraction; only the samples (iii) and (ii), located in the downstream section of the River Vilariça, showed higher clay and silt contents, 31% and 37.5%, respectively, in the WP sampling campaign (Fig. 2). In general, the sediments from the River Vizela were coarser, in particular in the Dry Period sampling; at the end of the Rainy Season, the finer fractions showed a slight increase. This trend is a consequence of the lithological parent material in the catchment area and the geomorphology of the river's longitudinal profile. In the River Vilariça, schist is the parent material, originating finer soil particles and the river has a lower gradient, more marked downstream in the large Vilariça valley; in the River Vizela, granite is the parent material, originating coarser particles arriving at the main course, and the geomorphology of the river longitudinal profile is steeper.

**Table 2:** Median, minimum and maximum values of the main sediment characteristics determined for granulometric fraction < 2 mm; metal concentrations in the granulometric fractions < 2 mm and < 63 µm (< d.l., < detection limit)

Granulometric fraction < 2 mm										Granulometric fraction < 2 mm					Granulometric fraction < 63 μm				
			pH	Coarse sand	Fine sand	Silt	Clay	OM	ECEC	Cd	Cu	Pb	Zn	Fe	Cd	Cu	Pb	Zn	Fe
					%				cmol(+) kg^-1^			mg kg^-1^					mg kg^-1^		
River Vilariça	Dry Period (DP)	median	6.4	76.7	16.9	3.0	3.4	0.8	4.4	10.6	119	18	107	20,579	8.9	222	39	142	27,698
		min.	6.1	68.9	4.9	1.5	2.7	0.2	2.7	8.3	99	16	87	14,179	5.1	103	24	107	25,516
		max.	6.4	90.2	22.9	4.2	4.1	1.0	6.3	17.4	162	27	119	27,058	17.8	341	55	241	32,260
	Wet Period (WP)	median	6.6	71.4	18.6	6.0	4.4	1.0	5.5	0.3	31	10	58	18,627	0.3	31	30	68	25,222
		min.	6.3	35.8	4.3	3.1	3.8	0.6	3.7	0.2	24	4	46	13,168	0.3	22	11	54	20,395
		max.	6.7	87.7	33.1	21.6	15.9	7.9	17.9	0.3	36	13	67	25,571	0.3	218	42	103	30,429
River Vizela	Dry Period (DP)	median	6.3	90.9	5.4	1.0	2.5	0.3	2.0	6.7	91	8	91	16,678	23.2	345	66	373	24,308
		min.	6.1	86.2	3.2	0.5	2.2	0.2	1.2	6.0	83	6	78	14,847	13.1	267	44	141	19,020
		max.	6.4	93.9	9.8	1.8	2.8	0.8	2.5	8.5	111	12	123	18,030	43.8	444	132	801	32,831
	Wet Period (WP)	median	6.7	76.0	17.9	2.9	3.9	0.9	3.0	< d.l.	11	4	55	14,596	15.9	142	31	131	16,600
		min.	6.5	39.3	11.2	1.7	3.2	0.7	1.7	< d.l.	9	1	45	11,793	8.1	18	20	36	14,005
		max.	6.9	82.8	48.5	6.6	5.6	2.1	4.9	< d.l.	20	6	86	18,655	40.8	168	42	181	19,328

Coarse sand (medium sand + coarse sand + very coarse): 2.0 – 0.250 mm;

Fine sand (very fine sand + fine sand): 0.250 – 0.063 mm;

Silt: 0.063 – 0.004 mm;

Clay < 0.004 mm

The sediment pH was slightly acidic in both river systems. The organic matter content of the granulometric fraction < 2 mm is low, varying in the range 0.2 to 2.1%. The contents are slightly in the River Vilariça and the samples (iii) and (ii), which showed higher clay and silt contents, also present higher OM contents, 5.9% and 7.9%, respectively. The OM contents are within the range of values obtained in other studies (e.g. Devesa-Rey et al. 2011; Mokwe-Ozonzeadi et al. 2019). The effective cation exchange capacity was slightly higher in the sediments from River Vilariça, around 4 cmol_(+)_kg^−1^, and the samples (iii) and (ii) also reveal higher values. In the River Vizela, sample (e) also showed a slightly higher value, as well as OM.

### Metal contents of the sediments

**Fig. 3:**
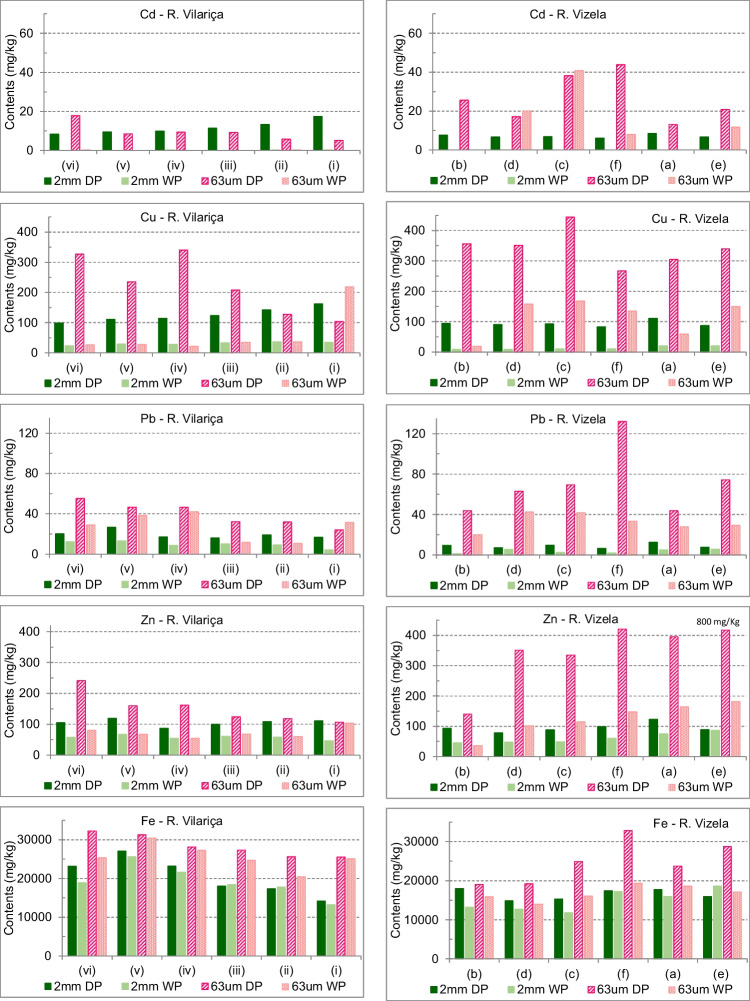
Distribution of spatial and temporal metal contents in the sediments of the River Vilariça and River Vizela, in the granulometric fraction < 2 mm and < 63 µm (sample site is indicated from upstream to downstream; DP – Dry Period; WP – Wet Period)

The studied metals’ contents for the granulometric fractions < 2 mm and < 63 µm are summarised in Table 2 and in Fig. 3, and, in general, the range of variation was higher in the River Vizela. In general, contents were higher in the granulometric fraction < 63 µm, and in the samples from the Dry Period. In the River Vilariça, Cd showed a different trend: the contents were slightly higher in the fraction < 2 mm, and it only occurred in the samples from the Dry Period (Fig. 3). In general, Cd, Cu, Zn, Pb, and Fe contents were higher in the more upstream sampling sites of the River Vilariça, (iv) to (iii), in particular in the granulometric fraction < 63 µm. This association may be a combination of the influence of the composition of the lithological parent material of the sediments and the use of chemical fertilizers in the olive grove and orchards.

The classification of the SQG (Sediments Quality Guidelines) of USEPA (MacDonald et al. 2000) establishes the PEL (Probable Effect Level) for Cd (3.53 mg kg^−1^), Cu (197 mg kg^−1^), Pb (91.3 mg kg^−1^) and Zn (315 mg kg^−1^). Based on this threshold values, Cd and Cu frequently showed contents above the PEL. Cadmium contents were, when occurring, above PEL in both fractions < 2 mm and < 63 µm. The range of variation was 5–18 mg kg^−1^ (DP), in the River Vilariça, and 13–44 mg kg^−1^ (DP) and 8–41 mg kg^−1^ (WP) in the River Vizela. Copper contents exceeded the PEL in the sediment fraction < 63 µm: in the River Vilariça, at the sample sites located more upstream (vi), (v), (iv), (iii), the values were in the range 208–341 mg kg^−1^ (DP); in the River Vizela, Cu contents showed a range of variation of 267–444 mg kg^−1^ (DP). In the River Vizela, Pb contents exceeded the TEL in sampling station (f); the Zn contents exceeded the PEL in all sampling stations, except (b), in the fraction < 63 µm of the River Vizela sediments, showing contents in the range 335–420 mg kg^−1^, with an anomalous high value of 801 mg kg^−1^, in sampling station (e), located near the confluence.

### Principal component analysis

The results of the principal component analysis on the metal contents and sediment properties, from each basin, yield 3 major components that explain 93% of the total variance of each dataset, from River Vilariça and River Vizela. The eighenvalues, explained variances, and the component loadings of the PCs with eighenvalues higher than 1 are given in Table S1, in the Supplementary Material. To assist data interpretation based on PCA the correlation matrices were used (Fig. S1 in the Supplementary Material).

The first component (PC1) explains 58 and 62% of the total variance in the datasets from River Vilariça and River Vizela, respectively, and represents the variation in the sediment granulometry, pH MO, ECEC and nutrients (N and P). Metals present negative loadings in this component. The second component (PC2) explains 25 and 24% of the total variance in the datasets from River Vilariça and River Vizela, respectively, and represents the variation in the metals. The third component (PC3) represents the variation of Fe in the River Vilariça, and the variation of OM and N in the River Vizela and explains 11% and 8%, respectively, of the total variance in the datasets.

### Enrichment of metal contents in the sediments

**Fig. 4:**
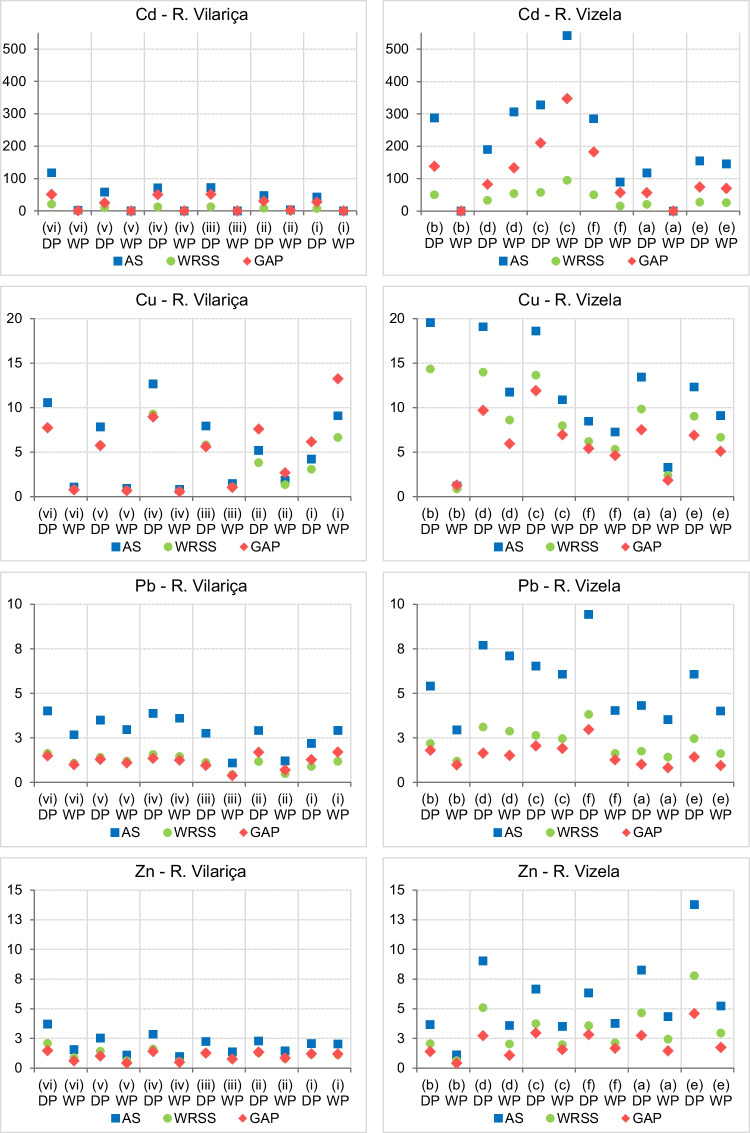
Enrichment factor (EF) for Cd, Cu, Pb and Zn in stream bed sediments from River Vizela and River Vilariça, using as background the average shale (Turekian and Wedepohl, in Salomons and Förstner,1984) – AS (  ), the world rivers suspended sediment (Viers et al 2009) – WRSS (  ), and Geochemical Atlas of Portugal (Ferreira 2000) – GAP (  ). Granulometric fraction < 63 µm. Sampling sites are indicated from upstream to downstream. DP – Dry Period; WP – Wet Period

The enrichment factors were calculated using Cd, Cu, Pb and Zn contents in the sediment fraction < 63 µm (Fig. 4). Similar trends for EFs of Cd, Cu, Pb, Zn were obtained when using the reference values from the average shale, world river suspended sediment or active streambed sediment from the Geochemical Atlas of Portugal. In general, the use of average shale concentrations increases the EFs for the metals under analysis. The results of EFs referenced to WRSS and GAP were very similar in the River Vilariça, except for Cd in the samples from the Dry Period, and Cu in sampling stations (ii) and (i), located more downstream, where the EFs referenced to GAP were the highest. In the River Vizela, the EFs referenced to the GAP were the lowest, except for Cd, where the EFs referenced to WRSS were the lowest. The calculation of EFs indicated that Zn and Pb present a minimal enrichment, with EFs ≤ 2; in the Vizela catchment the EFs were, in general, slightly higher indicating moderate enrichment, in particular in the Dry Period (2 ≤ EF ≥ 5). Regarding Cu, the EFs indicated significant enrichment, independently of the background used. The EFs of Cadmium indicated extremely high enrichment, with values surpassing 25 and a maximum of 348.

## Discussion

### Influence of sediment physico-chemical properties, OM and nutrients on the metal contents in sediments

Differences observed in metal contents between the two granulometric fractions were less pronounced in the River Vilariça samples. Besides, metal contents in samples from the Wet Period, < 2 mm, were higher than those observed in River Vizela. In this sampling campaign, Cu and Zn contents were similar at most sampling stations in both particle size fractions, except in site (i). These results suggest that in the River Vilariça, the lower fluvial competence, due to lower flows, leads to greater accumulation of sediments-bound metals in the riverbed, and a wider period of deposition and metal interaction with particles. In the River Vizela, part of the metal content in the fraction < 2 mm is probably retained in the finer particles. The higher flows and steeper channels lead to the washing of coarser particles and lower the time of residence of particles in the riverbed, decreasing the potential of interaction of metals with particle surfaces. Apparently, the effect of grain size is less significant on sediments’ metal contents from the River Vilariça.

The generally higher metal contents in the fraction < 63 µm is due to the well-known processes of adsorption/precipitation in, or within, the geochemical components of the water systems sediments: particles’ surfaces, organic matter, sulphides, Mn oxyhydroxides, amorphous and crystalline Fe oxides (Tessier et al. 1979; Salomons and Forstner 1984). During periods of low flow, mostly in the DP, the deposition of finer particles on the riverbed takes place, leading to an increase in the relative quantity of the finer fraction and associated metals in the riverbed material. In the WP, the metal contents decrease in both granulometric fractions, reflecting the dilution effect on the contaminant load from anthropogenic point sources of pollution, due to the higher water discharge. This will affect the partitioning between the dissolved and adsorbed phases, lowering the contaminants’ contents in the sediment compartment. On the other hand, the dilution effect caused by the increase in the grain size of deposited sediment and/or concomitant wash of finer particles also occurs. Soil particles dragged into the river network, carrying associated metals with them, during the rainy season and heavy precipitation events also contribute to the sediments’ metal contents (Ramos et al. 2015). In the River Vizela catchment there are more anthropogenic pressures, with consequently higher contaminant loads, resulting in higher metal contents in the fraction < 63 µm despite the higher flowing water dynamics.

**Fig. 5:**
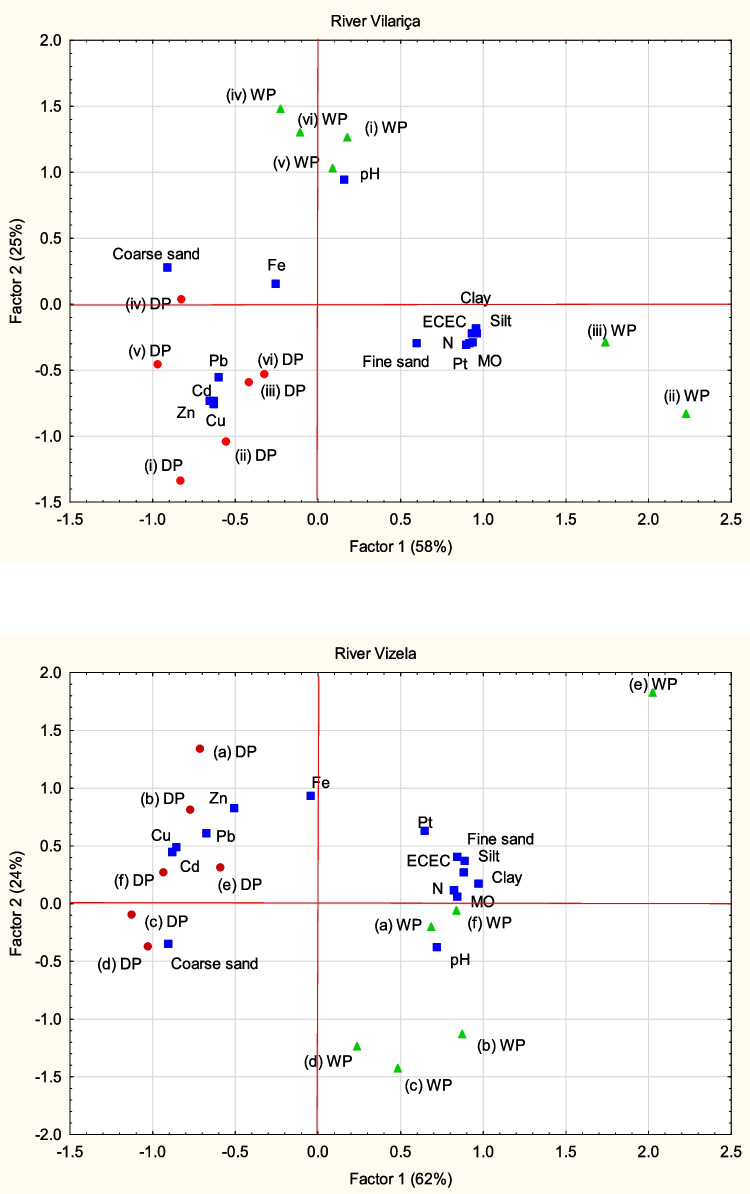
Projection of the component and samples score of the datasets from the Rivers Vilariça (**a**) and Vizela (**b**) onto the PC1-PC2 principal component plane

The OM and nutrient contents and the physico-chemical properties of sediment particles, such as particle size and ECEC, are considered major influential factors on the retention of metals in the aquatic environment (Forstner and Wittmann 1979; Horowitz 1991; Miranda et al. 2021a). Therefore, in order to explore the possible relationships among selected physico-chemical properties of sediments and metal contents, the PCA analysis was performed on the datasets from fraction < 2 mm. Figure 5 shows the projection of the components and case (samples) scores of the datasets from the Rivers Vilariça and Vizela onto the PC1-PC2 principal component plane (PC1 and PC2 amounted to 83 and 85% of data variance of River Vilariça and River Vizela, respectively). The samples from the DP and WP are discriminated in two clusters by the PC2 in the dataset from the River Vilariça, and by the PC1 in the dataset from the River Vizela.

The positive correlation between the finer fraction (silt and clay) and OM, ECEC, N and P, in PC1, is interpreted as the formation of organic coatings on the surface of the particles, and the existence of chemically active sites on the surface of the particles for cationic exchange. From Fig. 5, the samples (iii)WP and (ii)WP), from River Vizela, and (e)WP, (f)WP and (a)WP, from the River Vilariça, having higher contents of finer sediments show higher OM and associated ECEC (Fig. 2). The positive correlation between OM and N has is attributed to the presence of functional groups in the organic matter fraction. Phosphates associate by adsorption on the surfaces of sediment particles and Fe oxides and hydroxides (Evans et al. 2004; van der Perk et al. 2007; Withers and Jarvie 2008; Jan et al. 2015).

Metals display negative relationships with pH, particularly Cd, Cu and Zn. These results represent the influence of pH on the release of metals from sediments with the increase in concentration of H^+^ ions, which have strong exchange capacity and remove metals from negatively charged sites (Fairbrother et al. 2007; Miranda et al. 2021b). In the River Vizela these relationships are represented in the second component. In the River Vilariça the weaker relationship may be influenced by the low variability in pH values. Samples (f)WP (a)WP, and (i)WP, (iv)WP, (v)WP and (vi)WP reveal higher pH in sediments.

The weak-to-moderate negative correlation between OM, ECEC and metals, suggests that cation exchange reactions and OM play a minor role in the adsorption of metals to sediments. As reported in the literature, organic metal complexes are soluble and sensitive to pH reductions, which limits the adsorption of metals despite the interactions with organic ligands (Aiken et al. 2011). Besides, it is widely accepted that organic matter (mainly humic and tannin acids) plays a fundamental role in the process of metal adsorption in sediments of natural waters, particularly Cu (Forstner and Wittmann 1979; Ma et al. 2021). As mentioned by Mokwe-Ozonzeadi et al. (2019), the binding sites associated to the organic matter are related to the type of organic acid involved, for example, lignin has a lower surface binding, when compared to fulvic acids. The peaks of organic matter observed in Vilariça could be related to the effluents from a major wastewater treatment plant (WWTP) and several compact WWTPs dispersed in this part of catchment area, where the largest population centre (Vila Flor) and several villages are concentrated.

In the River Vizela, the PC2 represents the variation in P, Fe, Cd, Cu, Pb and Zn contents. The strong positive correlation of P with Fe may be attributed to binding of phosphorus compounds to Fe hydroxides (amorphous or with different degrees of crystallinity). Due to their large specific surface area and positive surface charge, pH dependent or not, Fe hydroxides have the ability to adsorb and bind phosphates. The second component, from both datasets, also shows moderate to strong positive contributions of metals. The projection of the components and samples (Fig. 5) show the clusters formed by metals and the samples from the Dry Period, representing the higher metal contents registered in the samples collected at the end of the Dry Period. The cluster formed by these metals suggest a similar source, which may be agriculture, urban activities, industry, or a combination of these (e.g. Alloway 2013; Miranda et al. 2022a). In the River Vizela, the association with P in the same factor, suggest the contribute from agriculture. The sampling at the WP took place after spring fertilizations. Frequently, this management practice involves the surface application of organic amendments from cattle production, which, when associated with events of intensive precipitation, contributes to the surface transport of considerable amounts of soil into the streams. The supply of sediments includes the input of metals and particulate P, as observed in other studies (e.g. van der Perk and Vilches 2020).

### Sediment quality assessment: comparative analysis of EFs by using different background values

The calculation of the EFs considered different background values: average shale, world rivers suspended sediments and the Geochemical Atlas of Portugal, given that no specific work has been carried out to determine background values for the studied catchments. In general, the use of average shale concentrations increases the EFs for the metals under analysis. The results of EFs referenced to WRSS and GAP were similar in the sediments from River Vilariça. In the River Vizela, the EFs referenced to WRSS were higher for Cu, Pb and Zn; for Cd, the EFs were higher when referenced to the GAP. Therefore, the results reveal an enrichment of Cu, Pb and Zn in the sediments of River Vizela, in relation to the contents presented by Viers et al. (2009) for suspended sediments of world rivers, even higher than EFs referenced to the GAP. It should be noted that the majority of world rivers are enriched in trace elements, compared with continental crust (Viers et al. 2009). Moreover, the metal contents from the GAP (Ferreira et al. 2001), although resulting from low density geochemical mapping, are most probably also enriched, due to historical industrial activity in the Vizela catchment (Brás 2019; European Parliament, Parliamentary question—E-004538/2021) and urban and agricultural pressures. There are no anomalous high metal contents recognised in the lithological units outcropping in the catchment area (Teixeira et al. 1973; Andrade et al. 1982). Cadmium is more enriched in relation to GAP, but in the study by Viers et al. (2009), it was found that the EF for Cd could be higher than 100.

Regarding the spatial distribution of EFs for Cd, Pb, Zn in the Vilariça catchment, these were higher in the sampling stations located more upstream and in the tributaries on the right margin, in areas covered by forest and cultivated areas. This trend is more marked in the Cd, Cu and Zn contents, which have the lithological contribution of the metasediments from the Ordovician and Silurian. In general, metasedimentary rocks contain higher contents of these metals (Reimann and de Caritat 1998), in relation to other lithologies outcropping in the area. Besides, Cd contamination is often associated to fertilizers in agricultural areas (Micó et al. 2006; N’guessan et al. 2009). Nevertheless, more research is needed regarding Cd since the contents are higher in the sediment fraction < 2 mm, which could be indicative of a significant contribution from the bedrock. Lead and Zn enrichments in forested areas have been attributed to atmospheric deposition trapped by canopies, with significance on a regional scale (Hernandez et al. 2003; N’guessan et al. 2009). Copper is more enriched downstream, in particular in the WP, an area occupied mainly by vineyards, where Cu-based fungicides have an intensive use (Droz et al. 2021). In the River Vizela catchment the lithological influence on the metal contents is not so evident.

When considering the sediment quality relative to the potential for adverse effects on sediment dwelling organisms, translated in the SQG classification, along with the EFs, it is observed that Cd is the metal showing higher risk, exceeding the PEL and presenting a very high to extremely high enrichment in the River Vizela (20 < EF > 542), and significant to extremely high enrichment (5 < EF > 118) in the River Vilariça DP. Copper is, in general, significantly enriched in the sediments (5 < EF > 20), except in the River Vilariça WP, but the Cu contents, in the River Vizela WP, are lower than the PEL. For Pb and Zn, in the River Vizela DP, there are samples that present contents above the PEL [(d), (c), (f), (a), (e)] and are classified as significantly enriched, if the EFs are referenced to the AS, and minimal to moderate enriched if the EFs are referenced to WRSS and GAP.

### Outcomes of the study in the context of implementation of WFD

The results of this study contribute with knowledge on the need of including sediments as a component in monitoring plans of aquatic systems quality, particularly in the implementation of the WFD. The above considerations on the data acquired for the two basins studied highlight the relevance of a geochemical approach when the water quality is under discussion. Sediments’ metal enrichment outputs through EF is a simplified and adequate methodological approach, although a proper geochemical background must be established.

This study highlights:

a) The use of the riverbed sediment fraction < 63 µm, in relation to the fraction < 2 mm, is advantageous, as similarities between the bed sediment fraction < 63 um and suspended sediment have been observed (Mokwe-Ozonzeadi et al. 2019), and it is the fraction that mainly constitutes the suspended load (Miller et al. 2015). Accordingly, data on metal contents in the finer fraction allows the comparison with databases on geochemical data of suspended sediments, more available worldwide than those of riverbed sediment. Besides, in dynamic mountain streams with steep gradient, the bed sediment collected in depositional areas retains recently deposited sediments and associated contaminants, which may provide information on possible pollution sources located upstream. The transport of this sediment fraction obeys to the cycle of deposition-resuspension controlled by streamflow velocity. Sediment grain size commonly affects trace metal concentration in river sediment. The silt–clay fraction, mostly composed of clay minerals, organic matter, and amorphous oxides of Fe and Mn, has high specific surface area and is geochemically active, thus having a high ability of retain metals (Forstner and Wittmann 1979; Horowitz 1991; Horowitz and Elrick 1988; Walling and Owens 2003; Förstner 2004; Collins and Walling 2007; van der Perk and Vilches 2020). High metal contents in finer sediment also affects benthic organisms that live in it and take food from it (Duan et al. 2009). One of the drawbacks in selecting this sediment fraction for environmental studies might be the amount of bulk sample needed in gravel bed rivers, with low proportion of finer sediment. Therefore, sediments sampling campaigns in rivers developed in mountainous catchments should be, preferentially, at the end of the dry period.

b) Considering both the natural geochemical background and EFs is significant in the assessment of environmental risks in freshwater systems. It may occur that low EFs associated with high natural enrichments correspond, in reality, to values of concern in terms of the sediment quality guidelines. The proper determination of background values would involve outlining the collection of an adequate set of samples of sediments in pristine streamlets. The proposal of Matys Grygar and Popelka (2016) on the use of empirical geochemical background functions, calculated from data on local unpolluted sediments, is, in our perspective, undoubtedly the adequate approach to evaluate natural vs. anthropogenic signals in sets of sediments of considerable size. However, this may not be a simple task as it seems. The selection of pristine sampling stations would have to take into account the combination of several elements such as geology, geomorphology and land use, in relation to upstream drained areas. From a scientific point of view, this might turn into a difficult task, especially in catchments with similar features to the ones considered in this study: relatively small drainage area, located in a mountainous region, which implies greater sedimentary dynamics, and a diversity of outcropping lithologies. The mineral associations originated within this framework are not constant in space and time. Moreover, old riverine sediments are difficult to find in such dynamic fluvial environments, and riverbanks, where sediment deposition is high, are often used for agricultural activities. Additionally, when there is a multitude of anthropic activities (e.g. livestock, agriculture, urban effluents, industry) dispersed over the catchments, these will imprint a non-natural signal to the geochemistry of local riverbed sediments, and finding recent sediments without anthropogenic contribution is almost impossible. In large rivers or marine sediments, the recovery of old, pre-impacted sediments is relatively simple, throughout the use of sediment core samples (Cruces 2015; Romano et al. 2015; Mil-Homens et al. 2013; Romano et al. 2022). Similar concerns about the determination of background values for riverbed sediments were mentioned by (e.g. Devesa-Rey et al. 2011). Furthermore, this type of work requires the availability of financial and human resources, which usually negatively affect its implementation, even in the simpler monitoring programmes. However, the construction of high-density geochemical maps, at least in areas of major environmental concern, could contribute to fulfil this gap, and once obtained it would support environmental issues.

The approach adopted in this study gave evidences about the useful information that sediment geochemistry contains, regarding the retention and transport of metals by regional mountain streams and the evaluation of the environmental quality of these fluvial systems. The assessment of metal enrichment in sediments can be used as an environmental indicator to address spatial and temporal variations of metal contamination in, and within, basins. This should be included in monitoring plans. Furthermore, the sediment quality criteria must be simple in the way it is transmitted to water quality managers. As emphasized by Meybeck (2013), “regional geochemical surveys provide a snapshot of sediment-associated metal contamination”, which is necessary to detect contaminant sources or identify lithological contributions.

## Conclusions

This study presents a discussion on metal contents in sediments collected in two river basins from the north of Portugal, through the calculation of EFs with reference to distinct background values. The EFs were higher when referenced to average shale concentrations. Regarding the background values derived from world rivers suspended sediments and the Geochemical Atlas of Portugal, the lower EFs should be interpreted with some reservation, since both backgrounds have anthropogenic contribution.

When the information on EFs was compared with the classification of metal contents based on SQGs, it was occasionally observed that sediments presenting contents above the PEL may be classified as minimal or moderate enriched, depending on the reference values taken for the calculation of EFs. Therefore, in the assessment of environmental risk in freshwater systems, EFs provide a simple classification of the quality of sediments with regard to metals, as long as the appropriate choice of reference values is made, preferentially the local, or regional, background contents supported by geochemical studies. Low EFs could be associated with natural enrichments and originate values of concern in terms of the sediment quality guidelines; on the contrary, high EFs may not necessarily represent risk to the fluvial environment.

For the effective integration of sediment quality in the monitoring plans, the communication of sediment quality to water quality managers must be relatively expeditious, and results should be presented in an easy reading and inter-comparable form. The calculation of metal enrichment in sediments is a useful technique, and relatively easy to apply, to distinguish between the geogenic and anthropogenic influence of metal contents in sediment, ensuring that the theoretical basis for its calculation are guaranteed. The identification of contamination (or natural enrichment) of metals in sediments can help decision-makers to support solutions for upstream pollution sources.

## Supplementary Information

Below is the link to the electronic supplementary material.

Supplementary file1 (PDF 209 KB)

## Data Availability

The data used in this study is presented in Supplementary Material.
