# Supplementary material for: Defining the quality of sediment in the context of the WFD monitoring plans: metal enrichment in two catchments from the north of Portugal
Source: J Soils Sediments. 2025 Feb 11;25(4):1373–91. doi: 10.1007/s11368-025-03963-6 (PMC12033116; doi:10.1007/s11368-025-03963-6)
Supplement: Supplementary file 1 — Supplementary file1 (PDF 209 KB) [file 11368_2025_3963_MOESM1_ESM.pdf]

Supplementary Information

Defining the quality of sediment in the context of the WFD monitoring plans: metal enrichment in two catchments from the north of Portugal

Anabela R. Reis[1]*, Bernardete Vieira[3], Marta Roboredo[2]

[1]Department of Geology, School of Life and Environmental Sciences, University of Trás-os-Montes e Alto Douro (UTAD), Quinta de Prados, 5000-801 Vila Real, Portugal; and CGeo - Geosciences Centre, University of Coimbra (Polo II), 3030-790 Coimbra, Portugal

[2]Department of Biology and Environment, School of Life and Environmental Sciences, and CITAB - Centre for Research and Technology of Agro-Environmental and Biological Sciences, CITAB, Inov4Agro, University of Trás-os-Montes e Alto Douro (UTAD), Quinta de Prados, 5000-801 Vila Real, Portugal

[3] Previous affiliation: Chemistry Center Vila Real, University of Trás-os-Montes e Alto Douro (UTAD), 5000-801 Vila Real, Portugal

* Corresponding Author: Anabela R. Reis, anarreis@utad.pt

Table S1 - The eighenvalues, explained variances, and the component loadings of the first 4 PCs for the datasets of River Vilariça and River Vizela.

Factor-variable correlations (factor loadings), based on correlations (**Vilariça** Seds <2mm in Seds 2mm correlações.stw)				
Variable	PC 1	PC 2	PC 3	PC 4
pH	0.16	0.94	0.15	0.24
Coarse sand	-0.91	0.28	0.08	-0.30
Fine sand	0.60	-0.30	-0.35	0.65
Silt	0.96	-0.19	0.17	-0.00
Clay	0.96	-0.22	0.11	-0.10
OM	0.94	-0.29	0.12	-0.12
ECEC	0.93	-0.23	0.24	0.01
N	0.92	-0.30	0.13	-0.22
Pt	0.90	-0.31	0.02	-0.13
Fe	-0.25	0.15	0.93	0.16
Cd	-0.65	-0.74	-0.15	-0.05
Cu	-0.63	-0.76	-0.13	-0.03
Pb	-0.60	-0.56	0.52	0.16
Zn	-0.63	-0.73	0.19	0.14

Factor-variable correlations (factor loadings), based on correlations (**Vizela** Seds <2mm in Seds 2mm correlações.stw)				
PC 1	PC 2	PC 3	PC 4	PC 5
0.72	-0.38	-0.39	-0.25	0.35
-0.90	-0.35	-0.02	0.19	0.15
0.89	0.37	-0.02	-0.21	-0.16
0.89	0.27	0.30	-0.16	-0.12
0.97	0.17	-0.01	0.05	-0.01
0.84	0.06	0.47	0.21	0.14
0.85	0.40	-0.25	0.10	0.09
0.83	0.11	0.51	0.09	0.18
0.65	0.63	-0.35	0.17	-0.03
-0.04	0.93	-0.27	0.17	0.02
-0.88	0.44	0.12	0.06	0.02
-0.86	0.49	0.11	0.00	0.02
-0.67	0.60	0.23	-0.27	0.11
-0.51	0.82	0.02	-0.12	0.15

Eigenvalues of correlation matrix, and related statistics (**Vilariça** Seds <2mm in Seds 2mm correlações.stw)				
Eigenvalue	8.09	3.45	1.49	0.74
% Total variance	57.82	24.61	10.64	5.27
Cumulative Eigenvalue	8.09	11.54	13.03	13.77
Cumulative %	57.82	82.42	93.06	98.33

Eigenvalues of correlation matrix, and related statistics (**Vizela** Seds <2mm in Seds 2mm correlações.stw)				
8.61	3.41	1.05	0.38	0.28
61.50	24.34	7.53	2.70	2.0
8.61	12.02	13.07	13.45	13.73
61.50	85.84	93.36	96.07	98.06

Figure S1 - Correlation matrix used in the PCA analysis

Correlations (Vilariça Seds 2mm in Seds 2mm correlações.stw) Marked correlations are significant at p < .05000 N=12

Variable	pH	Coarse sand	Fine sand	Silt	Clay	OM	ECEC	N	Pt	Fe	Cd	Cu	Pb	Zn
pH	**1.00**													
Coarse sand	0.06	**1.00**												
Fine sand	-0.08	-0.85	**1.00**											
Silt	-0.00	-0.91	0.57	**1.00**										
Clay	-0.06	-0.90	0.54	0.98	**1.00**									
OM	-0.14	-0.89	0.53	0.98	1.00	**1.00**								
ECEC	-0.03	-0.89	0.55	0.98	0.98	0.98	**1.00**							
N	-0.16	-0.84	0.46	0.95	0.99	0.99	0.95	**1.00**						
Pt	-0.16	-0.85	0.55	0.89	0.95	0.93	0.89	0.96	**1.00**					
Fe	0.27	0.30	-0.41	-0.11	-0.19	-0.19	-0.05	-0.20	-0.29	**1.00**				
Cd	-0.83	0.39	-0.15	-0.51	-0.48	-0.41	-0.48	-0.39	-0.37	-0.09	**1.00**			
Cu	-0.84	0.36	-0.13	-0.48	-0.45	-0.38	-0.44	-0.36	-0.33	-0.07	1.00	**1.00**		
Pb	-0.49	0.39	-0.27	-0.40	-0.41	-0.36	-0.32	-0.35	-0.35	0.55	0.70	0.72	**1.00**	
Zn	-0.72	0.34	-0.13	-0.43	-0.43	-0.37	-0.38	-0.36	-0.35	0.24	0.91	0.91	0.92	**1.00**

Correlations (Vizela Seds 2mm in Seds 2mm correlações.stw) Marked correlations are significant at p < .05000 N=12

Variable	pH	Coarse sand	Fine sand	Silt	Clay	OM	ECEC	N	Pt	Fe	Cd	Cu	Pb	Zn
**pH**	**1.00**													
**Coarse sand**	-0.51	**1.00**												
**Fine sand**	0.51	-1.00	**1.00**											
**Silt**	0.42	-0.95	0.93	**1.00**										
**Clay**	0.62	-0.93	0.92	0.89	**1.00**									
**OM**	0.40	-0.73	0.70	0.86	0.84	**1.00**								
**ECEC**	0.57	-0.87	0.87	0.77	0.88	0.66	**1.00**							
**N**	0.40	-0.75	0.72	0.87	0.82	0.98	0.63	**1.00**						
**Pt**	0.31	-0.77	0.78	0.60	0.77	0.45	0.89	0.44	**1.00**					
**Fe**	-0.31	-0.25	0.27	0.10	0.13	-0.07	0.41	-0.05	0.65	**1.00**				
**Cd**	-0.85	0.65	-0.63	-0.63	-0.79	-0.64	-0.57	-0.61	-0.32	0.41	**1.00**			
**Cu**	-0.83	0.60	-0.58	-0.59	-0.76	-0.64	-0.54	-0.60	-0.29	0.44	1.00	**1.00**		
**Pb**	-0.71	0.36	-0.35	-0.35	-0.55	-0.47	-0.41	-0.37	-0.17	0.48	0.87	0.88	**1.00**	
**Zn**	-0.60	0.17	-0.14	-0.21	-0.36	-0.37	-0.11	-0.31	0.14	0.77	0.80	0.84	0.87	**1.00**

Table S2 - Physico-chemical parameters, OM, P and metal contents of the sediments from the rivers Vizela and Vilariça (the results are the mean of 3 replicates). The coarse and medium sand fractions were grouped.

River Vilariça

Granulometric fraction <2 mm										Granulometric fraction <2 mm					Granulometric fraction <63 µm				
**Amostra**	**pH**	**Coarse and medium sand**	**Fine sand**	**Silt**	**Clay**	Silt+clay	**OM**	**ECEC**	**Pt**	**Cd**	**Cu**	**Pb**	**Zn**	**Fe**	**Cd**	**Cu**	**Pb**	**Zn**	**Fe**
		%	%	%	%		%	$cmol_{(+)}\ kg^{-1}$	mg kg-1	mg kg^{-1}	(mg/Kg)	(mg/Kg)	(mg/Kg)	(mg/Kg)	mg kg^{-1}	(mg/Kg)	(mg/Kg)	(mg/Kg)	(mg/Kg)
(vi) DP	6.4	68.9	22.9	4.2	3.9	8.2	0.9	6.3	376	8.3	99	20	105	23114	17.8	327	55	241	32260
(v) DP	6.4	89.0	4.9	3.0	3.0	6.0	0.6	5.4	254	9.4	111	27	119	27058	8.5	235	47	160	31268
(iv) DP	6.4	90.2	5.6	1.5	2.7	4.2	0.2	4.3	279	9.9	114	17	87	23204	9.3	341	46	162	28085
(iii) DP	6.4	71.6	20.8	3.9	3.7	7.6	0.8	4.5	345	11.4	123	16	100	18044	9.2	208	32	124	27311
(ii) DP	6.3	73.7	19.5	2.7	4.1	6.8	1.0	4.1	352	13.3	143	19	109	17323	5.7	128	32	119	25603
(i) DP	6.1	79.7	14.3	3.0	3.0	6.0	0.8	2.7	276	17.4	162	17	111	14179	5.1	103	24	107	25516
median	6.4	76.7	16.9	3.0	3.4	6.4	0.8	4.4	312	10.6	119	18	107	20579	8.9	222	39	142	27698
min.	6.1	68.9	4.9	1.5	2.7	4.2	0.2	2.7	254	8.3	99	16	87	14179	5.1	103	24	107	25516
max.	6.4	90.2	22.9	4.2	4.1	8.2	1.0	6.3	376	17.4	162	27	119	27058	17.8	341	55	241	32260
(vi) WP	6.7	81.4	11.2	3.5	3.9	7.4	0.6	3.7	358		24	12	57	18866	0.3	26	29	80	25350
(v) WP	6.7	69.8	17.5	7.8	4.9	12.6	1.1	6.1	295		29	13	67	25571		27	38	68	30429
(iv) WP	6.6	87.7	4.3	4.2	3.8	8.0	0.8	4.9	215	0.3	28	8	54	21616		22	42	54	27286
(iii) WP	6.5	35.8	33.1	19.1	12.0	31.1	5.9	16.2	545		33	10	61	18388		35	11	68	24709
(ii) WP	6.3	42.9	19.6	21.6	15.9	37.5	7.9	17.9	838	0.2	36	9	58	17809	0.3	36	11	60	20395
(i) WP	6.6	72.9	20.0	3.1	4.0	7.1	0.6	4.4	356	0.3	35	4	46	13168		218	31	103	25093
median	6.6	71.4	18.6	6.0	4.4	10.3	1.0	5.5	357	0.3	31	10	58	18627	0.3	31	30	68	25222
min.	6.3	35.8	4.3	3.1	3.8	7.1	0.6	3.7	215	0.2	24	4	46	13168	0.3	22	11	54	20395
max.	6.7	87.7	33.1	21.6	15.9	37.5	7.9	17.9	838	0.3	36	13	67	25571	0.3	218	42	103	30429

River Vizela

Granulometric fraction <2 mm										Granulometric fraction <2 mm					Granulometric fraction <63 µm				
**Amostra**	**pH**	**Coarse and medium sand**	**Fine sand**	**Silt**	**Clay**	Silt+clay	**OM**	**ECEC**	**Pt**	**Cd**	**Cu**	**Pb**	**Zn**	**Fe**	**Cd**	**Cu**	**Pb**	**Zn**	**Fe**
		%	%	%	%		%	$cmol_{(+)}\ kg^{-1}$	mg kg-1	mg kg^{-1}					mg kg^{-1}				
(b) DP	6.1	88.4	7.5	1.6	2.5	4.1	0.6	2.3	411	7.7	94	9	93	18030	25.6	356	44	141	19020
(d) DP	6.3	93.8	3.4	0.5	2.3	2.8	0.2	1.2	291	6.7	90	7	78	14847	17.1	352	63	351	19228
(c) DP	6.3	93.9	3.2	0.8	2.2	3.0	0.2	1.7	199	6.8	93	10	88	15320	38.2	444	69	335	24908
(f) DP	6.3	93.5	3.3	0.7	2.5	3.2	0.2	1.8	393	6.0	83	6	99	17411	43.8	267	132	421	32831
(a) DP	6.4	87.0	8.4	1.8	2.8	4.6	0.8	2.5	480	8.5	111	12	123	17730	13.1	305	44	396	23707
(e) DP	6.3	86.2	9.8	1.3	2.8	4.0	0.4	2.4	433	6.7	87	8	89	15945	20.8	339	74	801	28733
median	6.3	90.9	5.4	1.0	2.5	3.6	0.3	2.0	402	6.7	91	8	91	16678	23.2	345	66	373	24308
min.	6.1	86.2	3.2	0.5	2.2	2.8	0.2	1.2	199	6.0	83	6	78	14847	13.1	267	44	141	19020
max.	6.4	93.9	9.8	1.8	2.8	4.6	0.8	2.5	480	8.5	111	12	123	18030	43.8	444	132	801	32831
(b) WP	6.6	81.8	11.2	3.0	4.0	7.0	2.1	2.9	340		9	1	45	13217		18	20	36	15894
(d) WP	6.6	82.8	11.6	2.0	3.6	5.6	0.8	1.7	337		9	5	48	12689	20.1	157	42	102	14005
(c) WP	6.7	77.3	16.3	3.2	3.2	6.4	0.9	2.5	251		11	2	49	11793	40.8	168	42	114	16087
(f) WP	6.9	74.6	19.5	1.7	4.2	5.9	0.7	4.3	784		11	2	61	17237	8.1	135	33	147	19328
(a) WP	6.9	70.0	23.4	2.9	3.8	6.7	0.9	3.1	425		20	5	75	15976		59	28	164	18632
(e) WP	6.5	39.3	48.5	6.6	5.6	12.2	2.1	4.9	899		20	6	86	18655	11.7	150	29	181	17112
median	6.7	76.0	17.9	2.9	3.9	6.5	0.9	3.0	383		11	4	55	14596	15.9	142	31	131	16600
min.	6.5	39.3	11.2	1.7	3.2	5.6	0.7	1.7	251		9	1	45	11793	8.1	18	20	36	14005
max.	6.9	82.8	48.5	6.6	5.6	12.2	2.1	4.9	899		20	6	86	18655	40.8	168	42	181	19328

Coarse and medium sand: 2.0 - 0.250 mm;
Fine sand (very fine sand + fine sand): 0.250 - 0.063 mm;
Silt: 0.063 – 0.004 mm;
Clay < 0.004 mm.

River Vilariça

Granulometric fraction <2 mm										Granulometric fraction <2 mm					Granulometric fraction <63 µm				
**Amostra**	**pH**	**Coarse sand**	**Fine sand**	**Silt**	**Clay**	Silt+clay	**OM**	**ECEC**	**Pt**	**Cd**	**Cu**	**Pb**	**Zn**	**Fe**	**Cd**	**Cu**	**Pb**	**Zn**	**Fe**
		%	%	%	%		%	$cmol_{(+)}\ kg^{-1}$	mg kg-1	mg kg^{-1}	(mg/Kg)	(mg/Kg)	(mg/Kg)	(mg/Kg)	mg kg^{-1}	(mg/Kg)	(mg/Kg)	(mg/Kg)	(mg/Kg)
(vi) DP	6.4	68.9	22.9	4.2	3.9	8.2	0.9	6.3	376	8.3	99	20	105	23114	17.8	327	55	241	32260
(v) DP	6.4	89.0	4.9	3.0	3.0	6.0	0.6	5.4	254	9.4	111	27	119	27058	8.5	235	47	160	31268
(iv) DP	6.4	90.2	5.6	1.5	2.7	4.2	0.2	4.3	279	9.9	114	17	87	23204	9.3	341	46	162	28085
(iii) DP	6.4	71.6	20.8	3.9	3.7	7.6	0.8	4.5	345	11.4	123	16	100	18044	9.2	208	32	124	27311
(ii) DP	6.3	73.7	19.5	2.7	4.1	6.8	1.0	4.1	352	13.3	143	19	109	17323	5.7	128	32	119	25603
(i) DP	6.1	79.7	14.3	3.0	3.0	6.0	0.8	2.7	276	17.4	162	17	111	14179	5.1	103	24	107	25516
median	6.4	76.7	16.9	3.0	3.4	6.4	0.8	4.4	312	10.6	119	18	107	20579	8.9	222	39	142	27698
min.	6.1	68.9	4.9	1.5	2.7	4.2	0.2	2.7	254	8.3	99	16	87	14179	5.1	103	24	107	25516
max.	6.4	90.2	22.9	4.2	4.1	8.2	1.0	6.3	376	17.4	162	27	119	27058	17.8	341	55	241	32260
(vi) WP	6.7	81.4	11.2	3.5	3.9	7.4	0.6	3.7	358		24	12	57	18866	0.3	26	29	80	25350
(v) WP	6.7	69.8	17.5	7.8	4.9	12.6	1.1	6.1	295		29	13	67	25571		27	38	68	30429
(iv) WP	6.6	87.7	4.3	4.2	3.8	8.0	0.8	4.9	215	0.3	28	8	54	21616		22	42	54	27286
(iii) WP	6.5	35.8	33.1	19.1	12.0	31.1	5.9	16.2	545		33	10	61	18388		35	11	68	24709
(ii) WP	6.3	42.9	19.6	21.6	15.9	37.5	7.9	17.9	838	0.2	36	9	58	17809	0.3	36	11	60	20395
(i) WP	6.6	72.9	20.0	3.1	4.0	7.1	0.6	4.4	356	0.3	35	4	46	13168		218	31	103	25093
median	6.6	71.4	18.6	6.0	4.4	10.3	1.0	5.5	357	0.3	31	10	58	18627	0.3	31	30	68	25222
min.	6.3	35.8	4.3	3.1	3.8	7.1	0.6	3.7	215	0.2	24	4	46	13168	0.3	22	11	54	20395
max.	6.7	87.7	33.1	21.6	15.9	37.5	7.9	17.9	838	0.3	36	13	67	25571	0.3	218	42	103	30429

River Vizela

Granulometric fraction <2 mm										Granulometric fraction <2 mm					Granulometric fraction <63 µm				
**Amostra**	**pH**	**Coarse sand**	**Fine sand**	**Silt**	**Clay**	Silt+clay	**OM**	**ECEC**	**Pt**	**Cd**	**Cu**	**Pb**	**Zn**	**Fe**	**Cd**	**Cu**	**Pb**	**Zn**	**Fe**
		%	%	%	%		%	$cmol_{(+)}\ kg^{-1}$	mg kg-1	mg kg^{-1}					mg kg^{-1}				
(b) DP	6.1	88.4	7.5	1.6	2.5	4.1	0.6	2.3	411	7.7	94	9	93	18030	25.6	356	44	141	19020
(d) DP	6.3	93.8	3.4	0.5	2.3	2.8	0.2	1.2	291	6.7	90	7	78	14847	17.1	352	63	351	19228
(c) DP	6.3	93.9	3.2	0.8	2.2	3.0	0.2	1.7	199	6.8	93	10	88	15320	38.2	444	69	335	24908
(f) DP	6.3	93.5	3.3	0.7	2.5	3.2	0.2	1.8	393	6.0	83	6	99	17411	43.8	267	132	421	32831
(a) DP	6.4	87.0	8.4	1.8	2.8	4.6	0.8	2.5	480	8.5	111	12	123	17730	13.1	305	44	396	23707
(e) DP	6.3	86.2	9.8	1.3	2.8	4.0	0.4	2.4	433	6.7	87	8	89	15945	20.8	339	74	801	28733
median	6.3	90.9	5.4	1.0	2.5	3.6	0.3	2.0	402	6.7	91	8	91	16678	23.2	345	66	373	24308
min.	6.1	86.2	3.2	0.5	2.2	2.8	0.2	1.2	199	6.0	83	6	78	14847	13.1	267	44	141	19020
max.	6.4	93.9	9.8	1.8	2.8	4.6	0.8	2.5	480	8.5	111	12	123	18030	43.8	444	132	801	32831
(b) WP	6.6	81.8	11.2	3.0	4.0	7.0	2.1	2.9	340		9	1	45	13217		18	20	36	15894
(d) WP	6.6	82.8	11.6	2.0	3.6	5.6	0.8	1.7	337		9	5	48	12689	20.1	157	42	102	14005
(c) WP	6.7	77.3	16.3	3.2	3.2	6.4	0.9	2.5	251		11	2	49	11793	40.8	168	42	114	16087
(f) WP	6.9	74.6	19.5	1.7	4.2	5.9	0.7	4.3	784		11	2	61	17237	8.1	135	33	147	19328
(a) WP	6.9	70.0	23.4	2.9	3.8	6.7	0.9	3.1	425		20	5	75	15976		59	28	164	18632
(e) WP	6.5	39.3	48.5	6.6	5.6	12.2	2.1	4.9	899		20	6	86	18655	11.7	150	29	181	17112
median	6.7	76.0	17.9	2.9	3.9	6.5	0.9	3.0	383		11	4	55	14596	15.9	142	31	131	16600
min.	6.5	39.3	11.2	1.7	3.2	5.6	0.7	1.7	251		9	1	45	11793	8.1	18	20	36	14005
max.	6.9	82.8	48.5	6.6	5.6	12.2	2.1	4.9	899		20	6	86	18655	40.8	168	42	181	19328

Coarse sand (medium sand + coarse sand + very coarse): 2.0 - 0.250 mm;
Fine sand (very fine sand + fine sand): 0.250 - 0.063 mm;
Silt: 0.063 – 0.004 mm;
Clay < 0.004 mm.
